# Synthetic torpor protects rats from exposure to accelerated heavy ions

**DOI:** 10.1038/s41598-022-20382-6

**Published:** 2022-09-30

**Authors:** Anggraeini Puspitasari, Fabio Squarcio, Martina Quartieri, Cristina Totis, Timna Hitrec, Akihisa Takahashi, Yukari Yoshida, Kenji Hanamura, Tomoko Yako, Matteo Cerri, Palma Simoniello, Marco Durante, Walter Tinganelli

**Affiliations:** 1 GSI Helmholtzzentrum Für Schwerionenforschung GmbH, Planckstraße 1, 64291 Darmstadt, Germany; 2 Gunma University Heavy Ion Medical Center, Gunma 371-8511 Maebashi, Japan; 3 Department of Psychiatry, University of Wisconsin-Madison, Madison, WI USA; 4 Department of Physiology, Pharmacology, and Neuroscience, University of Bristol, Bristol, UK; 5 Department of Biomedical and NeuroMotor Sciences, University of Bologna, 40126 Bologna, Italy; 6 Istituto Nazionale Di Fisica Nucleare (INFN)–Sezione Di Bologna, 40126 Bologna, Italy; 7 Department of Pharmacology, Gunma University Graduate School of Medicine, Maebashi, Gunma 371-8511 Japan; 8 Department of Science and Technology, Parthenope University of Naples, 80133 Naples, Italy

**Keywords:** Biophysics, Cell biology, Physiology

## Abstract

Hibernation or torpor is considered a possible tool to protect astronauts from the deleterious effects of space radiation that contains high-energy heavy ions. We induced synthetic torpor in rats by injecting adenosine 5′-monophosphate monohydrate (5′-AMP) *i.p.* and maintaining in low ambient temperature room (+ 16 °C) for 6 h immediately after total body irradiation (TBI) with accelerated carbon ions (C-ions). The 5′-AMP treatment in combination with low ambient temperature reduced skin temperature and increased survival following 8 Gy C-ion irradiation compared to saline-injected animals. Analysis of the histology of the brain, liver and lungs showed that 5′-AMP treatment following 2 Gy TBI reduced activated microglia, Iba1 positive cells in the brain, apoptotic cells in the liver, and damage to the lungs, suggesting that synthetic torpor spares tissues from energetic ion radiation. The application of 5′-AMP in combination with either hypoxia or low temperature environment for six hours following irradiation of rat retinal pigment epithelial cells delays DNA repair and suppresses the radiation-induced mitotic catastrophe compared to control cells. We conclude that synthetic torpor protects animals from cosmic ray-simulated radiation and the mechanism involves both hypothermia and hypoxia.

## Introduction

Space radiation is generally acknowledged as one of the main health risks for human space exploration^1^. The majority of radiation dose absorbed by crews in manned interplanetary missions is produced by galactic cosmic radiation (GCR), high-energy charged particles, including densely ionizing heavy ions, produced in distant galaxies^2^. The energy of these particles is so high that shielding of the spacecraft cannot stop them and lead to exposure rates over 200 times higher than the radiation background on Earth^3^. For these reasons, radiation countermeasures for future missions, including drugs, dietary supplements, and novel shielding, are being investigated^4–7^.

Among the possible countermeasures, hibernation/torpor is becoming one of the most promising tools nowadays^8,9^. Hibernation is a special state used by some mammals (i.e., bears, squirrels), characterized by sequential episodes of torpor separated by brief interbout arousal, used to increase survival in harsh environments. During torpor, the metabolic rate is actively reduced by the animals and is followed by a decrease in body temperature proportional to the temperature gradient between the body and the environment^10,11^. During hibernation, the inactive animal is subject to dynamic physiological changes that lead to significant modification in tissue reaction, including acquired resistance to radiation damage^12,13^. In the early 60’s it was already noted that hibernating animals could survive a lethal dose of radiation and that post-irradiation duration of hypothermia positively correlated with survival^12–14^. These studies were eventually abandoned because no procedure to mimic torpor in humans was available.

Only recently the neural pathways that control torpor are being unraveled^15–17^. Moreover, it could also provide a much-needed method to improve the radiation resistance of space crews^18^. A condition mimicking hibernation which is known as synthetic torpor was successfully induced in non-hibernators such as rats by injecting the GABA_**A**_ agonist muscimol within the *raphe pallidus *area in the brain, a key thermoregulatory region^19^. Furthermore, rats undergoing this invasive treatment were shown to have an increased liver and testis radioresistance to X-rays^20^. Other studies showed that synthetic torpor is also inducible in rats by activating the central adenosine A1 receptors (A1ARs) using 5′-AMP^21,22^. This drug, even if administered immediately after radiation, showed to be effective in protecting mice against a TBI lethal dose of X-rays^23^. However, mice can enter torpor spontaneously, under the right set of circumstances, and it is now unknown if adenosine-mediated synthetic torpor can also protect non-hibernators, such as humans, from radiation damage. It is important to understand the protective effects against energetic heavy ions on non-hibernators as a simulation of the impact of cosmic radiation on astronauts during long-term missions.

Our hypothesis was that rats, a model of non-hibernating mammal, acquire resistance to high-energy heavy ions TBI when treated with 5′-AMP, inducing synthetic torpor. To simulate radiation quality in space, we resorted to (^12^C-ions) for TBI. To investigate the protective effect of adenosine-mediated synthetic torpor we analyzed liver and brain samples after irradiating the animals with a sub-lethal dose of ^12^C-ions. Moreover, we used investigated in vitro to clarify the mechanism of radioresistance. It has been argued that the lower concentration of oxygen in the tissues of an animal in synthetic torpor could be a relevant factor in preventing cell damage by radiation-induced ROS^24^. Furthermore, changes in the metabolism at low temperature may also have an impact on DNA repair^25^. To test these hypotheses, we irradiated rat retinal pigment epithelial (RPE-J) cells at reduced oxygen pressure and temperature with and without 5′-AMP.

## Results

### Synthetic hibernation may have protective effects on a lethal dose of C-ions

**Figure 1:**
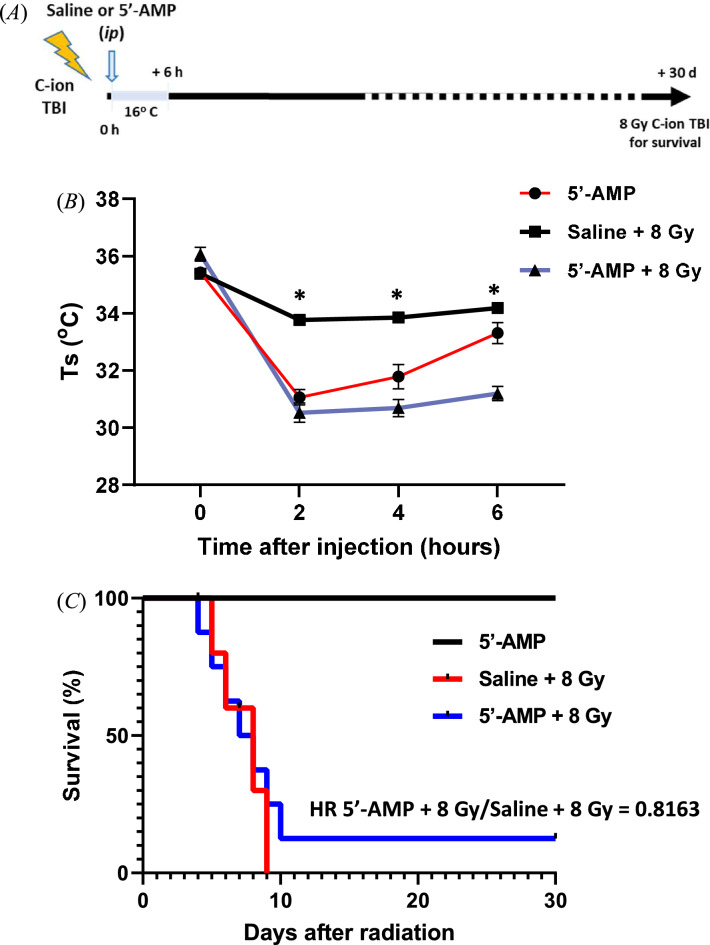
Synthetic hibernation induction in rats. (**A**) Experimental setup for radiation, hypothermia induction, and analysis. Total body irradiation (TBI). (**B**) The 5′-AMP administration, in combination with a low ambient temperature (Ta 16 °C), decreased their skin temperature (Ts) up to a moderate level of 27–32.9 °C (± 0.1–0.58) and (**C**) survival following 8 Gy C-ion whole-body irradiation, Hazard ratio (HR). Data are presented as the mean SEM. *P < 0.05 (Saline vs. 5′-AMP).

To investigate the survival of rats in synthetic hibernation, we irradiated male Sprague Dawley rats with a single dose of 8 Gy TBI followed by 5′-AMP via *i.p.* to induce synthetic torpor or saline injection as a sham control. Following injection, rats were exposed to a low-temperature room (+ 16 °C) immediately after irradiation for six hours. Figure 1B shows that in rats treated with 5′-AMP, skin temperature (Ts) dropped from ± 36 to 28–32.9 °C between 2 and 6 h after treatment. It showed that one hundred percent of saline-injected rats died between the first eight days, while 92% of 5′-AMP injected rats died within 11 days, and 8% survived over 30 days (Fig. 1C). The Cox-regression survival analysis shows no significant differences between the two Kaplan–Meier curves (*p* = 0.7767). Nevertheless, the hazard ratio is 0.8163 and is essentially caused by one rat in synthetic torpor that survived for the whole period of the irradiation. The statistic is insufficient to draw any firm conclusion, but seems to indicate that in some specific animals the response to synthetic torpor can be much stronger.

### Synthetic hibernation reduces the tissues damage of rats exposed to 2 Gy C-ion total body irradiation

**Figure 2:**
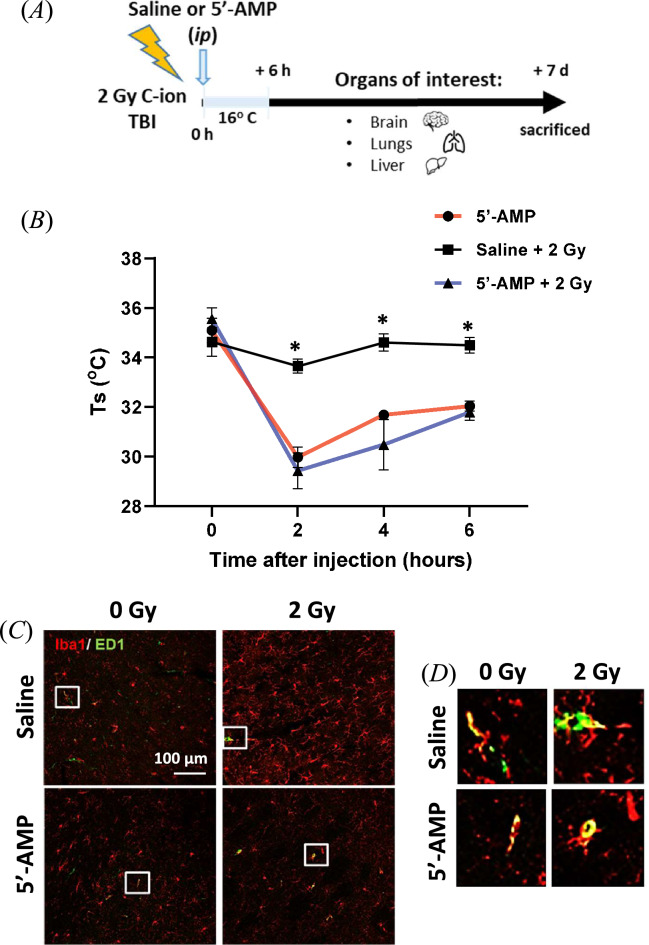

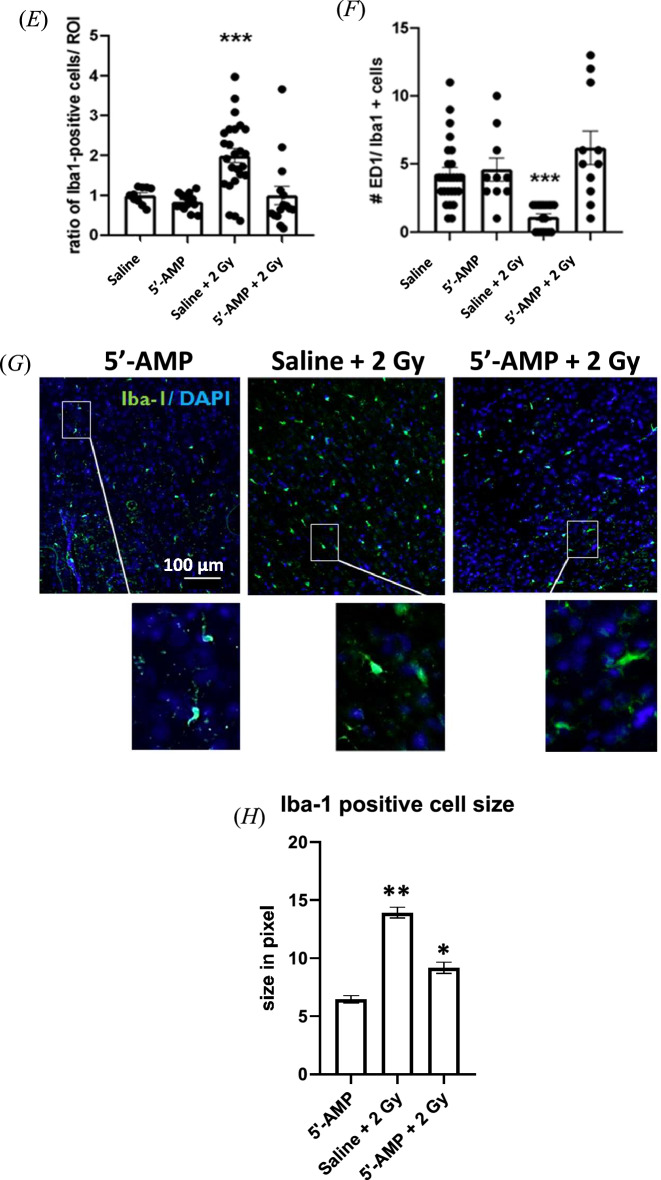
The 5′-AMP injection reduced activated microglia and maintained the number of resident macrophages. (**A**) Experimental setup for radiation, hypothermia induction, and analysis of the organs. (**B**) The 5′-AMP administration, in combination with a low ambient temperature (Ta 16 °C), decreased their skin temperature (Ts) up to a moderate level of 27–32.9 °C. (**C**) representative pictures and (**E**) the number and the size of Iba-1 positive cells (red) one week after 2 Gy of C-ions irradiation increased in rats treated with saline injection compared to the 5′-AMP treated and control non-irradiated rats (n = three rats; 5 slices from each rat). (**D**) Resident microglia with phagocytic function recognized as ED1 + /Iba-1 + (green/ red). (**F**) The 5′-AMP injected rats had the same number of ED1 + /Iba-1 + cells in control not-irradiated rats, while rats treated with saline injection significantly lowered the ED1 + /Iba-1 + cells one week after irradiation. (**G** and **H**) Irradiation increased the soma area of Iba-1 positive cells (green), while 5′-AMP injected rats had an increase compared to control animals and smaller size than saline-injected irradiated rats. Data are presented as the mean 6 SEM. *P < 0.05; **p < 0.01. Sham treatment, n = 12 slices (3 animals); 5′-AMP, n = 15 slices (3 animals); Saline + 2 Gy, n = 15 slices (3 animals); 5′-AMP + 2 Gy, n = 12 slices (3 animals).

To investigate the radioprotective effect in the organs of synthetic hibernation, rats were sacrificed one week after 2 Gy C-ion radiation exposure and tissues from brain, liver, and lungs were collected for immune-histological analysis (Fig. 2A). Following low-dose exposure in rats treated with 5′-AMP skin temperature (Ts) dropped from ± 36 °C to 29.4–32.0 °C.

#### Brain

The Iba-1 positive activated microglia are a marker of brain inflammation^26^. Histological examination of the brain’s sections shows a lower number of activated microglia cells after irradiation in the 5′-AMP treated rats compared to control. The number of Iba-1 positive cells, in the brain of the rats in synthetic torpor is comparable to the control animals (Fig. 2C,E). On the other hand, the numbers of Iba-1 positive cells after irradiation was significantly higher in rats at physiological temperature than in those under torpor (Fig. 2G,H). In addition, detection of the macrophage activation antigen ED1 was performed to understand the phagocytic activity of activated microglia. ED1 can be expressed on resident microglia or macrophage cells originating from the bone marrow. The resident microglia was recognized as ED1+/Iba-1+ positivity. These cells are responsible for myelin debris removal a critical function needed during injury’s recovery^27–29^. Results show that the ED1 + /Iba-1 + cells numbers in the 5′-AMP injected rats were comparable to the sham irradiated rats. On the other hand, in rats treated with saline injection the ED1+/Iba-1+ cells number significantly decreased one week after irradiation (Fig. 2D,F). These results indicate that the administration of 5′-AMP in combination with low temperature reduces radiation-induced brain inflammation.

#### Liver

**Figure 3:**
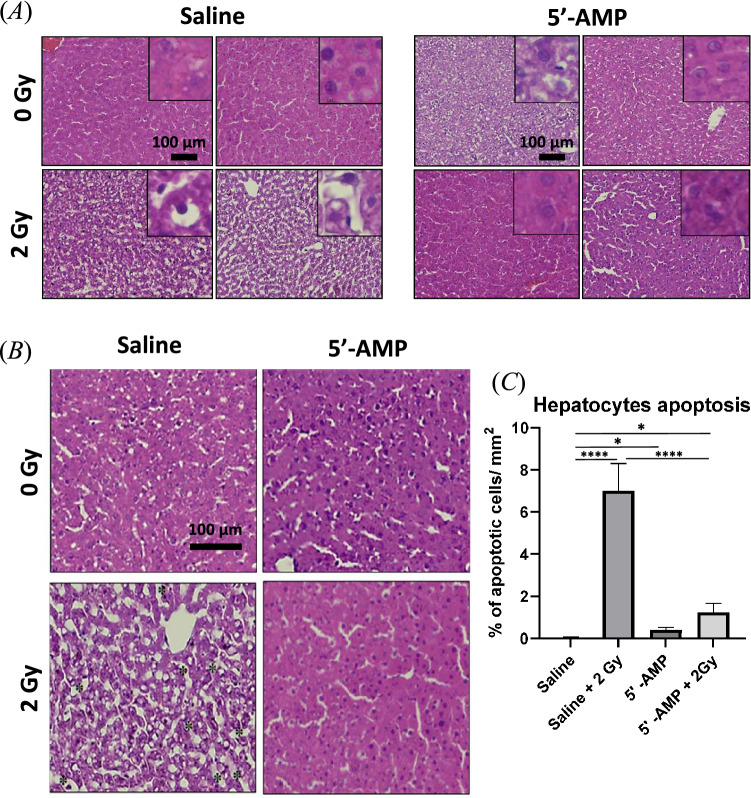
The 5′-AMP administration in rats suppresses the radiation-induced liver damage. (**A**) Irradiation-induced alteration in hepatocytes’ nuclei, cytoplasm (pyknosis). (**B**) Sinusoidal dilatation (asterisk) was observed on saline-injected irradiated rats, while the 5′-AMP administrated rats had preserved histology. (**C**) The increased number of pyknosis cells in the liver tissue of irradiated rats compared to those irradiated and treated with a 5′-AMP injection. Data are presented as the mean SEM. *P < 0.05; ****p < 0.0001. Sham treatment, n = 9 ROI (3 animals); 5′-AMP, n = 9 ROI (3 animals); Saline + 2 Gy , n = 9 ROI (3 animals); 5′-AMP + 2 Gy , n = 12 ROI (4 animals).

Histological analysis of the liver shows a radioprotective effect in the 5′-AMP injected rats compared to the saline-injected animals. In the liver of irradiated rats, alterations of nuclei and cytoplasm in the hepatocytes are evident. The hepatocytes have small nuclei, which is a typical feature of apoptotic or pyknotic cells, cytoplasms presents vacuolization and sinusoidal dilatation (Fig. 3A,B). Furthermore, disorganization of the parenchymal organization was observed. Quantitative analysis shows an increasing number of apoptotic cells in saline-injected rats, higher than in synthetic torpor rats (Fig. 3C). The sham irradiated rats treated with 5′-AMP present more apoptotic cells than the sham irradiated saline-injected rats’ liver, suggesting some toxicity of the 5′-AMP treatment.

#### Lung

**Figure 4:**
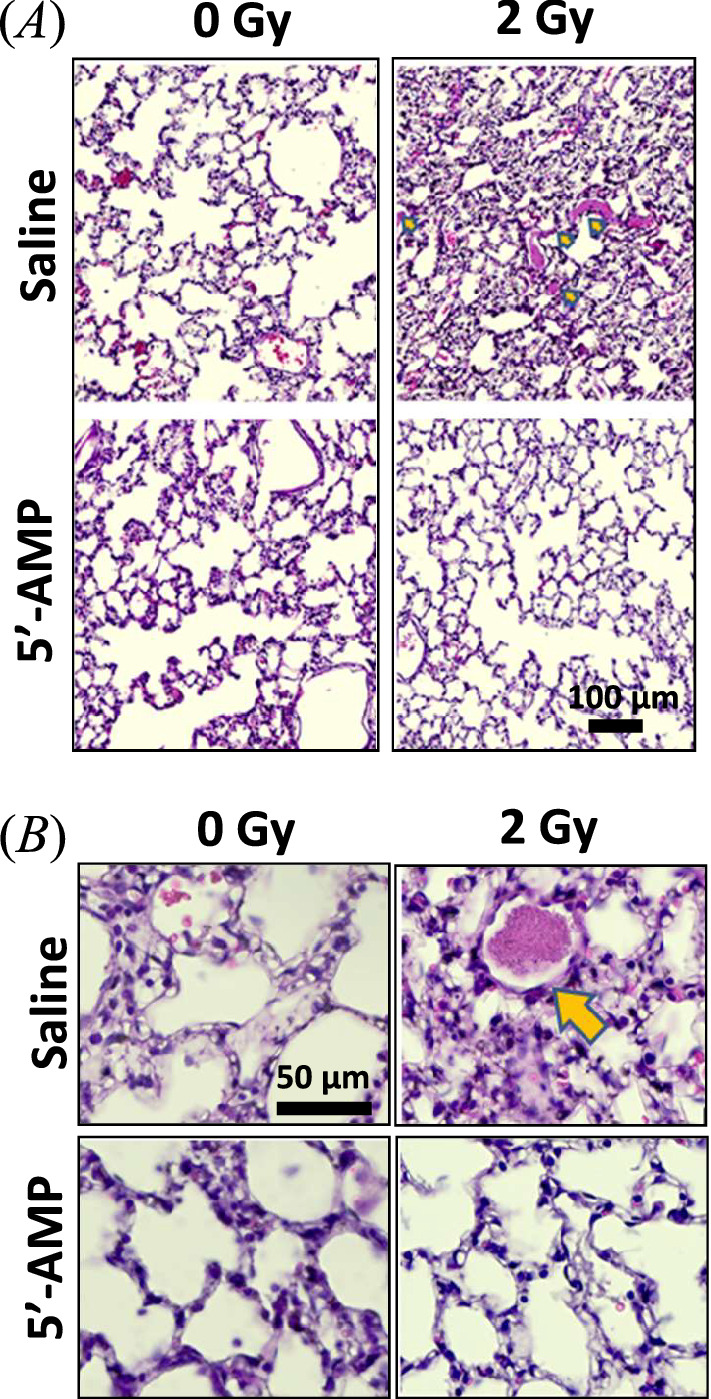
The 5′-AMP administration in rats suppresses the radiation-induced early damage to the lung tissue. (**A**) The H&E staining of lung tissues showed evident alteration one week after 2 Gy whole-body C-ions irradiation. (**B**) Proteinosis are observed in irradiated rats’ pulmonary alveoli, while the 5′-AMP treated rats have fewer proteinosis. Sham treatment, n = 3 animals; 5′-AMP, n = 4 animals; Saline + 2 Gy, n = 4 animals; 5′-AMP + 2 Gy, n = 4 animals.

The first stage of idiopathic pulmonary fibrosis (IPF) is visible in the lungs of irradiated rats. Histology of the lungs shows an evident alteration in the saline-injected irradiated animals compared to the rats in synthetic torpor (Figs. 2A, 4A). The changes in the lungs, such as alveolar composition and diffuse alveolar damage, including edematous alveolar septa, type II pneumocyte hyperplasia, and hyaline membrane formation, are similar to interstitial pneumonia^30^. Furthermore, a substantial accumulation of proteinosis into the alveolar space indicates a fibrotic process (Fig. 4B). To confirm the proteinosis accumulation and to distinguish from the red blood cells accumulation, the periodic acid Schiff (PAS) staining analysis was performed (supplementary data). The lung histology results showed that radiation damages were reduced in the rats injected with 5′-AMP.

### Radiation-induced foci and mitotic catastrophe

**Figure 5:**
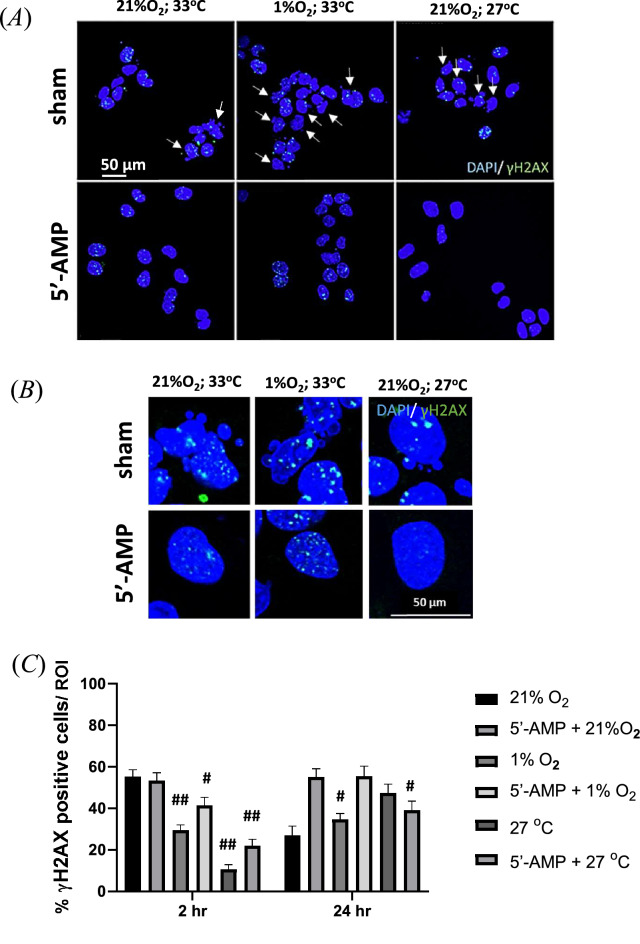
The 5′-AMP delays the radiation-induced DNA repair and decreases the mitotic catastrophe after irradiation in RPE-J cells. (**A**) Representative images of γH2AX foci (green) positive cells inside the nucleus (DAPI, blue). The irradiated cells without 5′-AMP showed irregular nuclei (arrow point). (**B**) In the magnifying images of nuclei morphology, the sham cells showed catastrophic mitotic features. (**C**) The percentage γH2AX positive nuclei. A significant decrease up to 30 to 40% of γH2AX positive nuclei (< 10 foci) observed on cells kept in a hypoxic even lower number on low temperature, less than 20% with and without 5′-AMP treatment. Twenty-four hours after irradiation, the γH2AX positive nuclei (< 10 foci) showed a reduction to 30% in normoxic conditions without 5′-AMP. The cells that were maintained in hypoxia before are comparable to normoxic cells. The cells were kept in the low-temperature condition before showing higher positive nuclei than 2 h results. Data are presented as the mean SEM. #*P* < 0.001; ##*p* < 0.0001. Images were obtained from 10 to 25 random ROIs (two coverslips from each experiment).

In vitro experiments using an established rat cell line retinal pigment epithelial cells (RPE-J) were performed to investigate the mechanisms of the effect observed in vivo. To understand whether the 5′-AMP molecule treatment combined with hypoxia or low temperature was involved in radiation protection, we added the 400 µM of 5′-AMP molecule to the cell medium immediately after 2 Gy of C-ion irradiation. Cells were cultivated at 27 °C (low-temperature), with 21% O_2_ or at 33.6 °C, with 1% O_2_ concentration (hypoxia); or 33.6 °C, with 21% O_2_ (normoxia) for six hours. The DAPI staining shows that 5′-AMP treatment may suppress the radiation-induced mitotic catastrophe of the cells (Fig. 5A,B). Two hours after radiation, there is no difference in the number of γH2AX foci for the cells cultivated in normoxia independently from the 5′-AMP administration. The percentage of γH2AX foci positive cells at 24 h, which are maintained in hypoxia, was similar to sham-treated cells 24 h after irradiation (Fig. 5A,C). The increased foci 24 h in cells with 5′-AMP is suggestive of a delay in DNA repair. It does not has an effect on mitotic catastrophe though, which is indeed reduced at 24 h compared to control cells after irradiation.

## Discussion

Previous studies have already shown the successful attempt of using the 5′-AMP molecule for inducing synthetic torpor or a hypometabolic state in mouse, an animal that can undergo spontaneous torpor^21,23,31^. Our current study showed the possibility of inducing synthetic torpor in rats, a non-hibernator, 5′-AMP via *i.p.* in combination with low temperature (+ 16 °C) (Figs. 1B, 2B). It has been shown that hibernation decreases the tissue’s oxygen demand, and those changes may lead to tissue hypoxia, a condition that increases tissue radioresistance^32–34^. Those physiological changes were induced by the administration of 5′-AMP (+ 16 °C) and lead to better protection to the organs after C-ions irradiation (Figs. 2, 3 and 4).

In the current study, survival analysis after 8 Gy of C-ion irradiation shows no significant differences. However, we observed radiation effects one week after 2 Gy of C-ion irradiation and found there is an increased number of activated microglia following radiation and the number of activated cells was found to be significantly suppressed by the 5′-AMP administration (Fig. 2C,E). Our findings suggest that the 5′-AMP treatment can suppress the number and size of microglia or Iba-1 positive cells induced by radiation (Fig. 2C,E,G and H) and maintain macrophage activity which are recognize by ED1/Iba-1 positive cells (Fig. 2D,F). This maintenance of macrophage activity by 5′-AMP induced torpor may mitigate the radiation-induced brain injury due to the importance of this phagocytic function^35^.

In liver, we observed a significant increase of apoptotic hepatocytes, cell vacuolization, and dilatation of the sinuses’ parenchyma one week following 2 Gy TBI while the rats treated with 5′-AMP have shown fewer apoptotic hepatocytes and preserve their liver histology well (Fig. 3). It has been reported that within 48 h the pro-inflammatory cytokines play an important role in radiation-induced liver disease^36^ and the study from Zhan et al.^37^ showed that 5′-AMP could inhibit pro-inflammatory cytokines in D-galactosamine/lipopolysaccharide-induced liver mice model. A similar pathway might inhibit radiation induced pro-inflammatory cytokines and lead to the suppression of pathological phenotypes in the livers one week after irradiation of the rats injected with 5′-AMP.

Furthermore, our current study observed changes in the lungs one week after irradiation. In our study, the acute change in the lungs, such as protein accumulation within alveoli, seems to start one week after 2 Gy of C-ion irradiation and the 5′-AMP treatment is able to suppress the protein accumulation (Fig. 4 and Supplementary Fig. 1). These acute changes are mostly asymptomatic as the symptoms of radiation-induced liver and lung injuries occur later^38,39^. The alteration of the inflammatory cytokines and apoptosis has been identified in the lung parenchyma within a few hours after injury^40^. Those early alterations might impair pulmonary surfactant homeostasis and lung immune function and lead to pulmonary alveolar proteinosis^41^.

It has been demonstrated that adenosine is an important immunomodulatory^42,43^ and the application of 5′-AMP on rats may act as extracellular adenosine signaling functions to prevent excessive inflammation by suppressing proinflammatory cytokines^43,44^. Although the 5′-AMP is known as a non-selective A1 receptors agonist^45,46^, it have shown to regulate the lymphocytes through the adenosine A2 receptors and the activation of the A3 receptors^42,47^. Those mechanisms of action of 5′-AMP in regulating the immune system might lead to the protective effects against radiation-induced lung and liver injury.

It is also necessary to understand whether 5′-AMP treatment on cells acts directly on the cells without immune intervention; therefore, we carried out the in vitro model. It is known that hypoxia is a significant reason for the resistance of tumor cells to radiation and the radiosensitivity of cells depends on the oxygen time deprivation after X-rays and C-ion irradiation^48^. In current study, we showed that two hours following irradiation under hypoxia and low-temperature conditions, the radiation-induced phosphorylation of γH2AX has reduced even without 5′-AMP treatment (Fig. 5A). However, 24 h after irradiation, the γH2AX in control-irradiated cells had already decreased, in contrast the cells treated with 5′-AMP and in hypoxia and low temperature for six hours showed a significant increase. It seems that the DNA damages repair is being delayed by 5′-AMP treatment under the low oxygenation or temperature level. Interestingly, despite the high number of γH2AX positive cells 24 h after irradiation, the 5′-AMP treated cells do not present radiation-induced mitotic catastrophe like those without 5′-AMP treatment (Fig. 5B). 5′-AMP is known as activator of a class of protein kinases known as AMP-activated protein kinase (AMPK)^49^. The metabolic adaptations by 5′-AMP treatment through AMPK as an energy sensor under conditions of ATP depletion, such as hypoxia and inhibition of oxidative phosphorylation^50^, might save the cells from radiation induced mitotic catastrophe.

In conclusion, synthetic torpor generated by 5′-AMP showed radiation protection effects on the organs such as brain, lungs and liver after C-ions irradiation. Moreover, despite the complexity and the homeostasis of the living animal body system, the in vitro study showed that the radiation protection effects derive from a combination of metabolic adaptation induced by 5′-AMP, hypoxia and low-temperature conditions.

## Methods

### Ethical Approval

All animal experiments were performed according to guidelines set by the Animal Care and Experimentation Committee of Gunma University, Showa Campus, Maebashi, Japan. Every effort was made to minimize animal suffering and the number of animals used and complied with the ARRIVE guidelines. All experimental protocol were approved by Committee Animal Care and Experimentation, Gunma University, Showa Campus, Maebashi, Japan (Approval Number: 19-095).

### Animals

Six to seven-week-old male Sprague Dawley (250 ± 15 gr) were used (SLC Co., Ltd., Shizuoka, Japan). The animals were kept at Experimental Animal Facility at an ambient temperature (T_a_) and relative humidity of 23–25 °C and 55% respectively. Unlimited food and tap water was provided ad libitum, and the animals were maintained on a 12 h light and 12 h dark cycle.

### In vivo study

After one week of habituation, total body irradiation was given to the with a dose of 2 or 8 Gy of C-ions at Spread Out Bragg Peak (SOBP) with energy 290 MeV/u. Immediately after whole-body irradiation, rats were either treated with vehicle (phosphate-buffered saline) or 5′-AMP (900 mg/kg; Sigma-Aldrich, St. Louis, MO, USA) via intraperitoneal injection (*i.p*). Following the injection, rats were kept for six hours in a 16 °C cold room. The skin temperature (T_s_) of the animals was measured using the Thermal infrared camera (FLIR E5xt (incl. Wi-Fi) Serial Number (S/N) 639,072,792) every two hours and for six hours after saline or 5′-AMP injection. After six hours, the animals were returned to the home cage at room ambient temperature and observed after they recovered. The animals irradiated with 2 Gy were sacrificed one week following the irradiation. The organs such as; brain, liver, and lungs were collected for the immune and histological analysis. Animals irradiated with 8 Gy were used for the 30 days survival study. After irradiation, animals were kept for 30 days at room temperature (Fig. 1A). The number of animals used for 2 Gy irradiation study are as follow: sham saline n = 3; 5′-AMP inj. n = 4; Saline + 2 Gy IR n = 5; 5′-AMP + 2 Gy IR n = 5. For survival after 8 Gy irradiation study, sham saline n = 6; 5′-AMP n = 5; Saline + 8 Gy IR n = 8; 5′-AMP + 8 Gy IR n = 12.

### In vitro study

The retinal pigmented epithelium (RPE-J) normal cells from rats were used for the γH2AX foci and morphology of nuclei. RPE-J cells were purchased from the American Type Cell Culture Collection (ATCC, Manassas, VA, USA). Cells were harvested in T75 flasks and maintained at 33.6 °C in a 21%O_2_ incubator, as suggested by the ATCC, to preserve the phenotype of the cells. One day before irradiation, cells were subcultured on the 80 mm coverslips inside a 12-well plates. Immediately after 2 Gy of C-ions irradiation, three wells of 12 well plates for each condition were treated either with DMSO or 400 µM of 5′-AMP, as previously described. Two of 12-well plates for each condition were kept at 33.6 °C in the 21% O_2_; 33.6 °C 1% O_2_; and 27 °C 21% O_2_ to incubate for six hours. After six hours, the medium was taken out, and cells were washed with warm PBS, replaced with a new medium, and returned to the 33.6 °C, 21%O_2_ incubator. For immunocytochemistry during the drug incubation, the cells were fixed two hours after irradiation. After the drug was washed out with PBS, the cells were fixed twenty-four hours after irradiation for γH2AX staining.

### C-ions radiation setup

C-ions were generated using 290 MeV/u, with 150 mm × 150 mm irradiation for in vivo irradiation. The rats were placed side by side in a fixed tube and irradiated two animals at once. The irradiated animals received a single absorbed dose of 2 Gy or 8 Gy. Sham-irradiated animals were transported to the radiation facility, injected with saline, and put in the same 16 °C cold room but not exposed to radiation. While for in vitro study, a single dose of 2 Gy of C-ions was used. The cells in 12 well plates and T25 flasks were irradiated vertically with the minimum medium during the irradiation.

### Immunohistochemistry

The impact of irradiation on the brain, liver and lungs of 2 Gy irradiated rats was examined. We irradiated 5–6 rats per group, and three rats per group were used for analysis (4–7 slices from each animal). For the immunohistochemically analysis, the brains were removed and post-fixed in 4% paraformaldehyde in PB at 4 °C for 24 h and then transferred to 30% sucrose in PB. After equilibration, 30-µm-thick coronal sections of the brain were cut [− 3.64 mm to − 1.24 mm from the bregma, using a rat brain atlas as reference] using a cryostat (CM1860 UV, Leica, Wetzlar, Germany). The sections were washed in PBS, incubated with 0.1% Triton X-100 for 15 min, and blocked for 30 min in 3% bovine serum albumin in PBS (PBSA). Sections were then incubated overnight with the primary antibody at 4 °C. After the sections were washed in PBS, they were incubated with the secondary antibody for one hour, rinsed with PBS, and then mounted.

The brain sections were analyzed using a confocal laser scanning microscope. Images were obtained with a Confocal fluorescence Leica microscope (Leica, Germany) using Leica automation and image analysis software (Meta Imaging V7.7; RRID: SciRes 000,136; Molecular Devices, Sunnyvale, CA). Images were acquired at an excitation wavelength of 568 nm for Iba-1 and 488 nm for activated ED1, then with an ultraviolet laser for DAPI. Each region of interest (ROI) were selected randomly and observed at 341 nm/pixel (1024 × 768 pixels) with a 10 × objective (numerical aperture, 0.70). The images used for the comparison in this study were collected under identical conditions, and the images were processed using MetaMorph software (Meta Imaging Software Version 7.7; MetaMorph Microscopy Automation and Image Analysis Software, RRID: SciRes_000136; Molecular Devices, Sunnyvale, CA). The intensities of Iba-1 and ED1 were analyzed from four random regions of interest (ROI) in layers 2–5 of the cerebral cortex. The number of positive Iba-1 and or ED1 cells was quantified. For each group, 4 to 5 slices of the brain from each animal were used. Sham treatment, n = 12 slices (3 animals); 5′-AMP , n = 15 slices (3 animals); Saline + 2 Gy , n = 15 slices (3 animals); 5′-AMP + 2 Gy , n = 12 slices (3 animals).

### Haematoxylin–Eosin staining

The liver and lungs were fixed in 10% formalin neutral buffer solutions for one day and then stored in 70% ethanol at 4 °C upon paraffin embedding process. Then specimens were embedded in paraffin wax after the fixation. Sections of liver and lung tissues samples were cut into 5 um thickness with a microtome (Leica RM2125, Germany) and mounted on a glass slide and were then deparaffinized in xylene and rehydrated in a graded ethanol series then stained with haematoxylin–eosin (HE staining) to analyse their morphology. Periodic acid-Schiff staining (PAS staining) to observe the protein accumulation on the alveoli of the lungs. Liver and lungs sections images were obtained using ZEISS Axio Scan.Z1 Digital Slide Scanner (Carl Zeiss Microscopy, NY) then analysis was performed using QuPath version 0.2.3. software. The images were analyzed from three random regions of interest (ROI) in the liver and lungs of each animal. Sham treatment, n = 3 animals; 5′-AMP , n = 4 animals ; Saline + 2 Gy , n = 4 animals; 5′-AMP + 2 Gy , n = 4 animals.

### Antibodies

The following primary antibodies were used: for microglia, marker using rabbit anti-Iba1 monoclonal (1:200; abcam, Cambridge, UK); for ED1 using mouse anti-CD68 monoclonal (1:100; Bio-Rad, Hercules, CA); anti Phospho histone H2A.X (Ser139) mouse monoclonal antibody (1:250). The following secondary antibodies were used for immunohistochemistry: Alexa 488 goat anti-mouse IgG (1:500; Molecular Probes, Eugene, OR); Alexa 488 goat anti-rabbit IgG (1:500; Molecular Probes, Eugene, OR); Alexa 647 goat anti-mouse IgG (1:500; Molecular Probes, Eugene, OR). Counterstaining was performed with DAPI (1:1000; Cellstain Dojindo, Japan).

### Statistical analysis

Comparisons between two groups were performed using the unpaired t-test. Comparisons between more than two groups were performed using analysis of variance followed by Dunnett’s test using GraphPad Prism 9.

## Supplementary Information

Supplementary Information.

## Data Availability

The datasets used and/or analysed during the current study available from the corresponding author on reasonable request.
