# Supplementary figures and images for: Synthetic torpor protects rats from exposure to accelerated heavy ions
Source: Sci Rep. 2022 Sep 30;12:16405. doi: 10.1038/s41598-022-20382-6 (PMC9525701; doi:10.1038/s41598-022-20382-6)

**Supplementary Figure 1.**  
**PAS staining**

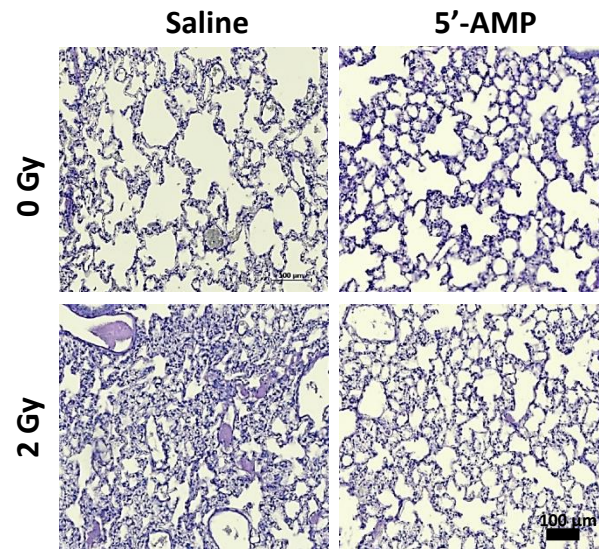

Supplement: Supplementary file 1 — Supplementary Information. [file 41598_2022_20382_MOESM1_ESM.pdf]
